# Iowa Medical Journal

**Published:** 1867-12

**Authors:** 


### EDITORIAL.

In presenting the Profession with the first No. of the re-issue of the Iowa Medical Journal, I deem it due them, as well as myself, to state that the present number is only a fair sample in size and style of workmanship. The matter is not such as we hope to fill its pages with hereafter; and instead of the large share of advertising which we present you, we will promise you a full share of reading and readable matter. We will continue with each number to publish some valuable articles from the members of our State Medical Society, and keep you posted with what is going on in the medical world, both at home and abroad. Will promise you, if nothing better offers, a synopsis, at least, of my journeyings, last year, through portions of the Old World: a bird's-eye view of their colleges, hospitals, modes of teaching; manners, customs and colors of those taught; their great professional lights, in contrast with those of our own country; will go with you to Rome, to Naples, Mount Vesuvius, Herculaneum, and Pompei; show you the head of a Cæsar, and the vertebræ of a Monk, and, if I dare, would tell you how secured, and where they came from.

